# 3-(2-Bromoethyl)-indole inhibits the growth of cancer cells and NF-κB activation

**DOI:** 10.3892/or.2015.3970

**Published:** 2015-05-11

**Authors:** KHALDA FADLALLA, RAMY ELGENDY, EBONY GILBREATH, SATYANARAYANA R PONDUGULA, TESHOME YEHUALAESHET, MAHMOUD MANSOUR, TESFAYE SERBESSA, UPENDER MANNE, TEMESGEN SAMUEL

**Affiliations:** 1 Department of Pathobiology, College of Veterinary Medicine, Nursing and Allied Health, Tuskegee University, Tuskegee, AL, USA; 2 Department of Pharmacology, Faculty of Veterinary Medicine, Suez Canal University, Ismailia, Egypt; 3 Department of Anatomy, Physiology and Pharmacology, College of Veterinary Medicine, Auburn University, Auburn, AL, USA; 4 Department of Natural Sciences, Elizabeth City State University, Elizabeth City, NC, USA; 5 Department of Pathology and Comprehensive Cancer Center, University of Alabama at Birmingham, Birmingham, AL, USA

**Keywords:** cancer, indole, 3-(2-bromoethyl)-indole, bioactivity, NF-κB, small-molecule inhibitor

## Abstract

Indole-3-carbinol (I3C) and diindolylmethane (DIM), found in cruciferous vegetables, have chemopreventive and anticancer properties. In the present study, 14 substituted indoles were tested for activity against SW480 colon cancer cells. Among these, 3-(2-bromoethyl)-indole, named BEI-9, showed the greatest inhibition. The effects of BEI-9 on cancer cells were analyzed by MTS and CellTiter-Glo assays for effects on cell viability, by microscopy for phenotypic changes, by scratch wound assays for effects on migration, by flow cytometry for changes in the cell cycle, by immunoblotting for cyclin D and A to assess effects on cell cycle regulation, and by NF-κB reporter assays for effects on basal and drug-induced NF-κB activation. BEI-9 inhibited the growth of SW480 and HCT116 colon cancer cells at concentrations of 12.5 and 5 *µ*M, respectively. BEI-9 also inhibited cell motility as determined with scratch wound assays, and reduced the levels of cyclin D1 and A. Furthermore, in reporter cells, BEI-9 (0.8 *µ*M) inhibited basal and induced NF-κB activation and increased cell death when combined with the cytokine TNFα or the drug camptothecin (CPT), both of which activate NF-κB. Preliminary experiments to identify a safe dose range for immunodeficient mice showed that BEI-9, administered intraperitoneally, was tolerable at doses below 10 mg/kg. Thus, BEI-9 and other indole derivatives may be useful in chemoprevention or as chemosensitizers. Since NF-κB activation is implicated in carcinogenesis and in reducing sensitivity to anticancer drugs, BEI-9 should be investigated in combination with drugs such as CPT, which activate NF-κB.

## Introduction

The indole chemical moiety is a backbone for several bioactive compounds including the amino acid tryptophan, the signaling compound melatonin, the nutritional compound indole-3-carbinol (I3C), and several receptor or kinase agonists or antagonists. I3C, abundant in cruciferous vegetables, is suggested to be one of the most active ingredients responsible for the anticancer benefits of such vegetables (1,2). Upon exposure to acidic environments, such as that in the stomach, I3C is converted into derivatives with variable stability and bioactivity (3). Diindolylmethane (DIM), an acid condensate of I3C, is thought to be one of the bioactive derivatives (4). I3C has antimicrobial activity against *Staphylococcus*, *Enterococcus*, *Escherichia*, and *Pseudomonas* microorganisms (5).

Indole compounds have potential as chemopreventive and chemotherapeutic agents. Cellular targets identified for natural or derivatized indole compounds include PPARγ and Nur77 (6), transcription factors (7–10), cyclin-dependent kinase (CDK) complexes (11,12), PKB/Akt (13,14), and hormone receptors (15,16). Indole compounds have activity against colon cancer cells, suggesting their potential use in chemoprevention or therapy (17–20). A full description of cellular targets and potential mechanisms of actions of indole compounds is available (21,22).

Despite the biological relevance of indole compounds, the bioactivities of many indole derivatives, especially those related to I3C, remain unknown. To evaluate the activities of indoles with a structural relationship to I3C, 14 compounds were selected from an indole library and their effects were tested on cells derived from human colon cancers. After an initial screening of these at 50 µm, BEI-9 was identified as a potent inhibitor of cell proliferation. We also identified BEI-9 as an inhibitor of the NF-κB signaling pathway at submicromolar concentrations. A preliminary test to determine a safe dose to mice showed that BEI-9 could be administered at doses below 10 mg/kg without obvious pathological changes or toxicological signs. These results suggest that BEI-9 and its derivatives or analogues could be developed into bioactive drug entities.

## Materials and methods

### Cell culture

SW480 and HCT116 cells were purchased from the American Type Culture Collection (ATCC) and maintained in McCoy’s 5A medium containing antibiotics and fetal bovine serum (FBS). Luciferase reporter cells were generated and used for experiments as described previously (23). HepG2 human liver carcinoma cells were obtained from the ATCC and grown in dulbecco’s modified Eagle’s medium (DMEM) supplemented with 10% FBS, 100 U/ml penicillin, 100 *µ*g/ml streptomycin, 2 mM L-glutamine, and 1 mM sodium pyruvate. The assay media included phenol red-free DMEM supplemented with 5% charcoal/dextran-treated FBS and the other additives. All cells were cultured in an incubator with a humidified atmosphere under 5% Co_2_ and 95% air at 37°C.

### Chemicals and plasmids

Dimethyl sulfoxide (DMSO), rifampicin, and SR12813 were purchased from Sigma-Aldrich (St. Louis, MO, USA). pcdna3-human pregnane X receptor (hPXR) and pGL3-CYP3A4-luc plasmids were as previously described (24,25).

### hPXR transactivation assays

HepG2 cells were transfected with pGL3-CYP3A4-luc reporter and pcDNA3-hPXR plasmids using FuGENE 6 (Promega, Madison, WI, USA). After 24 h of transfection in growth media, 10^4^ cells in the assay media were plated into 96-well culture plates (PerkinElmer) and exposed to DMSO (vehicle) or a PXR agonist, rifampicin or SR 12813, for an additional 20 h. At 10 min before the luciferase activity assay using the Neolite Reporter Gene Assay system (PerkinElmer), DMSO or BEI-9 (10 *µ*M) was added to the cells, which were incubated at 37°C and room temperature for 5 min each. Luminescence was measured with a FLUOstar Optima microplate reader (BMG Labtech).

### MTS and CellTiter-Glo assays

[3-(4,5-Dimethylthiazol-2-yl)-5-(3-carboxymethoxyphenyl)-2-(4-sulfophenyl]-2H-tetrazolium (MTS) cell proliferation assay and CellTiter-Glo^®^ Luminescent Cell Viability Assay kit (Promega) were used, according to the manufacturer’s instructions, to evaluate the viability of cancer cells. For both assays, 10^4^ cells/well of 96-well plates were exposed to the test compounds for 24 or 48 h, after which the assays were performed. Readings from vehicle-treated cells were used to normalize the data. The results were expressed as viability indices representing relative percentages compared to the controls. For experiments with HepG2 cells, cells in the assay media were plated into 96-well culture plates (PerkinElmer) at a density of 10^4^ cells/well and exposed to DMSO or a PXR agonist (rifampicin or SR12813) for 20 h. BEI-9 (10 *µ*M) was added to the cells 10 min prior to measuring the luminescence with the CellTiter-Glo luminescent assay system and a FLUOstar Optima microplate reader.

### Microscopy

Phase-contrast images of cells were captured at x20 magnification (and a 10x eyepiece) using an Olympus IX71 inverted microscope fitted with a digital camera equipped with CellSens^®^ Image Capture software (Olympus America, Inc., Center Valley, PA, USA). Images were stored in TIFF format and subsequently cropped and resized using Microsoft PowerPoint.

### Cell cycle analysis

Cells were prepared for flow cytometry as described previously (26). Cells were harvested by trypsinization with 0.25% trypsin-EDTA (Invitrogen Corp., Carlsbad, CA, USA) and then centrifuged. Pellets were suspended in 300 *µ*l of phosphate-buffered saline (PBS; Invitrogen Corp.) and fixed by addition of 700 *µ*l of 100% ethanol while vortexing. Next, the cells were stored at −20°C for a minimum of 12 h. Fixed cells were centrifuged and stained in FACS staining solution (320 mg/ml RNase A and 0.4 mg/ml propidium iodide) in PBS without calcium and magnesium. Stained cells were filtered through 70-*µ*m filters and analyzed by flow cytometry on a C6 Accuri^®^ flow cytometer (Accuri Cytometers, Ann Arbor, MI, USA). Data were analyzed, and histograms were prepared using CFlow™ software (Accuri Cytometers).

### Scratch wound assays

Cells were grown to confluency in 6-well plates, and the monolayers were wounded by scratching the layer of cells with the tip of a 200-*µ*l pipette. Scratch positions were microphotographed every 24 h for 96 h. At 48-h intervals, depleted culture medium was replaced with fresh medium containing the same concentration of BEI-9. Widths of the wound gaps were measured by an electronic micrometer scale, and the results were plotted on a graph.

### NF-κB reporter luciferase assays

For luciferase assays, cells were seeded and treated in 96-well plates. Before reading the plates, the culture medium was removed by aspiration, and 50 *µ*l of 1X luciferin-PBS substrate solution was added to each well. With a luminometer set at 37°C, plates were read immediately after addition of substrate solution and subsequently after 5 and 10 min. The time-points at which peak readings for the wells were obtained were selected for calculation of relative luciferase units (RLU). Luciferase expression was quantified as RLU, normalized to readings of control wells, and expressed as relative NF-κB reporter activity.

### Immunoblotting

Cell lysates were prepared in RIPA cell lysis buffer (Sigma-Aldrich) containing a protease inhibitor cocktail. Protein concentrations were determined using a detergent-compatible protein assay (Bio-Rad Laboratories Inc., Hercules, CA, USA). Samples containing equivalent protein concentrations were mixed with Laemmli’s buffer and boiled for 5 min. Proteins were resolved by SDS-PAGE, transferred to PVDF membranes (GE Healthcare Life Sciences, Piscataway, NJ, USA), and blocked in 5% non-fat dry milk. Primary antibodies for cyclin A (Upstate Biotechnology, Lake Placid, NY, USA) and cyclin D (Millipore, Darmstadt, Germany) were used at 1:1,000 dilutions. Mouse anti-tubulin antibody was purchased from Sigma-Aldrich. Peroxidase-conjugated anti-rabbit and anti-mouse IgG secondary antibodies were purchased from GE Healthcare Life Sciences and used at 1:5,000 dilutions. Chemiluminescent detections were performed with Classico or Crescendo Premixed Chemiluminescent HRP substrates (Millipore, Billerica, MA, USA).

### Experiments with mice

All animal procedures were approved by the Tuskegee University Animal Care and Use Committee. As a prelude to performing anticancer studies on BEI-9, a safe-to-administer dose for nude mice was determined by intraperitoneal injection of BEI-9 to nude mice at 6 weeks of age. To prepare the injections, volumes of BEI-9 stock solution were mixed with sterile PBS to a final volume of 1 ml. Solutions (100 *µ*l) containing the desired BEI-9 concentrations were injected. As controls, DMSO injections were prepared similarly. Portions of these solutions prepared for injections were saved to determine if the amounts used for injection of mice were effective *in vitro*. Initially, small numbers of mice were injected once with a dose of 1, 50, or 100 mg/kg of BEI-9. After elimination of single injections of 50 and 100 mg/kg doses due to toxic outcomes, additional mice were administered the vehicle or 1, 5, or 10 mg/kg of BEI-9 once a week for 6 weeks. Body weights and general conditions of health were followed to assess any unusual effects of the treatment.

## Results

### BEI-9 inhibits cell proliferation

**Figure 1:**
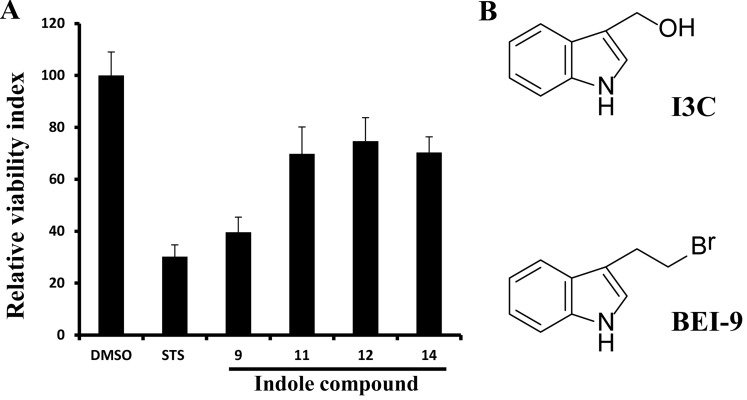
The relative bioactivities of BEI-9 and three other indole compounds. (A) SW480 colon cancer cells were exposed to a 50 *µ*M concentration each of indole compound 9 (BEI-9), 11, 12 or 14. MTS assays were performed to measure cell viability after 24 h of treatment. (B) Structures of indole-3-carbinol (I3C) and BEI-9.

A subset of 14 compounds (Table I) from a commercial indole library (Sigma-Aldrich) was screened by use of cell viability assays. To measure their bioactivities, a starting concentration of 50 *µ*M was tested. Two independent assays for cell viability, MTS assays and CellTiter-Glo assays, were performed. The MTS assay measures a reduction in tetrazolium dye by NAD(P) H-dependent oxidoreductases, whereas the CellTiter-Glo assay is dependent on the relative amount of ATP available in the cells, which is required for the activity of the enzyme luciferase to catalyze a luminescent reaction. BEI-9 was the most potent, with activity greater than I3C. In the 24-h assay (Fig. 1A), BEI-9 reduced the proliferation index of SW480 cells by ~60%, relative to 30% for I3C and was chosen for further testing. The structures of I3C and BEI-9 are shown in Fig. 1B.

**Figure 2:**
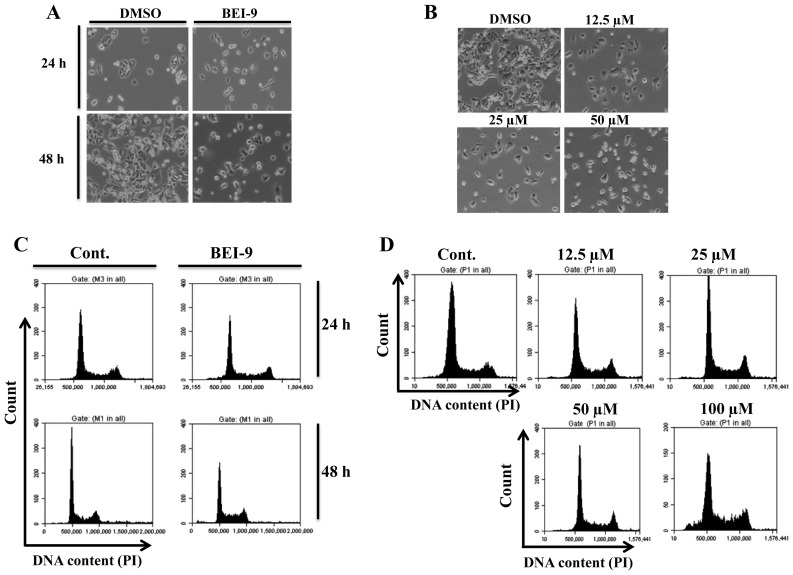
BEI-9 is an inhibitor of cell proliferation without induction of apoptosis. (A) SW480 colon cancer cells were exposed either to dimethyl sulfoxide (DMSO) as vehicle control or BEI-9 (50 *µ*M). Phase-contrast images were captured at 24 (upper panels) or 48 h (lower panels). (B) SW480 colon cancer cells were exposed to DMSO as a vehicle control or to 12.5, 25, or 50 *µ*M concentrations of BEI-9. Phase-contrast images of the cells were captured at 48 h after treatment. (C) Cell cycle of cells treated as in panel a, showing no induction of a sub-g1 population of cells, indicative of apoptosis. (d) Cell cycle profiles of cells exposed to the indicated concentrations of BEI-9 showing a small induction of sub-G1 cells only at a high concentration (100 *µ*M). The x-axes of the histograms in both panels C and D indicate propidium iodide (PI) staining intensity, representing the DNA content of cells, whereas the y-axes indicate cell counts.

To determine whether the reduction in the proliferation index by BEI-9 was due to cell death or to decreased proliferation, cells were seeded in 24-well dishes at 10^4^ cells/well and exposed to either the vehicle (DMSO) or BEI-9 (50 *µ*M). Phase-contrast images of the cells were recorded at 24 and 48 h after treatment. The monolayers were not washed, in order to preserve any dead cells. BEI-9-treated SW480 cells failed to proliferate, but vehicle-treated cells continued to proliferate, an effect most evident at 48 h (Fig. 2A). Since there was no increase in the number of floating (detached or dead) cells, BEI-9 apparently blocked SW480 cell proliferation without causing cell death. A dose-response experiment involving concentrations ranging from 12.5 to 50 *µ*M indicated that amounts <50 *µ*M inhibited cell proliferation (Fig. 2B). Furthermore, the possibility of inhibited cell proliferation without death of SW480 cells was assessed by cell cycle analysis of cells exposed to BEI-9 (50 *µ*M) for 24 or 48 h. In agreement with the phenotypic observation, the cell cycle profiles of BEI-9-treated cells were indistinguishable from the control cells at 24 and 48 h after treatment (Fig. 2C). SW480 cells were also exposed for 24 h to BEI-9 concentrations ranging from 12.5 to 100 *µ*M, and their cell cycle profiles were analyzed by flow cytometry. Evidence for increased cell death was evident only at 100 *µ*M (Fig. 2D). Since such a high concentration is unlikely to be achieved in animals, the primary mechanism of action of BEI-9 apparently does not involve induction of cell death.

### BEI-9 inhibits cell motility in wound scratch assays

**Figure 3:**
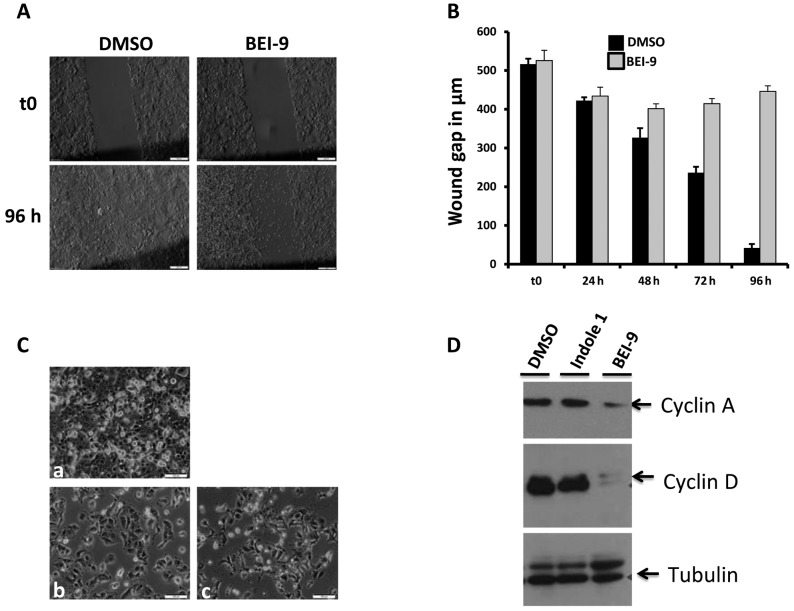

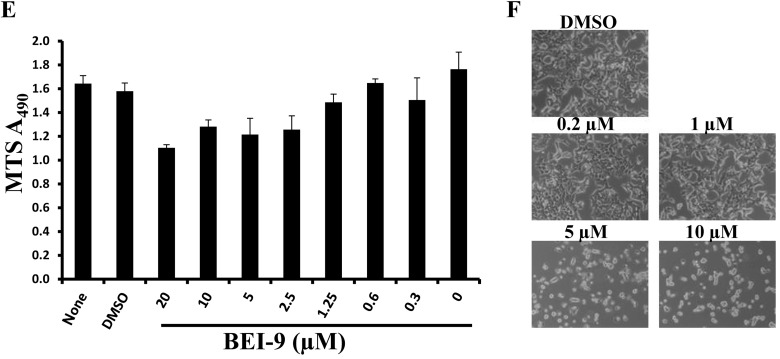
Effects of BEI-9 on motility, survival, recovery from treatment, and cyclin levels in treated cells. (A and B) The scratch wound assay was performed as described in Materials and methods. Phase contrast images of the wounded and then dimethyl sulfoxide (DMSO) or BEI-9 treated SW480 cell monolayers were taken at 24-h intervals. Panel a shows images of the same spots taken immediately after scratching (upper panels) and then 96 h after the first treatment (lower panels). Panel B shows widths of scratch wound gaps measured at 24-h intervals for 96 h. (C) Sparsely seeded SW480 cell monolayers were exposed to either DMSO or BEI-9 (25 *µ*M) for 48 h. Then the monolayers were washed with culture medium and further incubated for up to 4 days. DMSO-treated cells became 100% confluent within 24 h of washing (a), whereas BEI-9-treated cells did not recover from the treatment, as shown on micrographs at the wash time (b) or 4 days after washing (c). (D) SW480 cells were exposed to DMSO, indole 1 (25 *µ*M, inactive), or BEI-9 (25 *µ*M) for 24 h. Protein levels of cyclin A or D were detected by immunoblotting. Tubulin served as a loading control. (E and F) Effects of BEI-9 on HCT116 colon carcinoma cells. The viability of cells exposed for 24 h to the indicated concentrations of BEI-9 was measured with the MTS assay. Absorbance values at 490 nm wavelength are shown on the y-axis (E). Photomicrographs of cells exposed for 48 h to DMSO or BEI-9 (0.2, 1, 5, or 10 *µ*M) are shown (F).

Since BEI-9 is an inhibitor of cell proliferation, its effects on cell motility, as determined by a scratch wound assay, were assessed. SW480 cells were grown to 100% confluency and wounded by scratching the monolayers with a pipette tip. Cells were exposed to BEI-9 (25 *µ*M) or DMSO, the vehicle. Healing of the wound by migration of cells from the wound edge was followed by imaging at 24-h intervals. The DMSO-treated cells migrated, closing the wound gap within 96 h (Fig. 3A and B). In contrast, BEI-9-treated cells failed to close the gap. Thus, BEI-9 inhibited the migratory capacity of these cells.

### SW480 cells fail to recover from the effect of BEI-9

Since the phenotypic outcome of treating SW480 cells with BEI-9 was to ‘freeze’ the cells at the status quo at the time of treatment, it was possible that the cells, after a period of exposure, could recover if the agent was removed from the culture medium. SW480 cells were exposed to BEI-9 (25 *µ*M) and left in the medium for 48 h. After the treatment, cell monolayers were washed 5 times with regular growth medium, and then left in the same medium for up to 4 days. Vehicle-treated cells grew to confluency in 48 h, but BEI-9-treated cells did not recover from the treatment even at 4 days after withdrawal of the drug (Fig. 3C). Although the cells did not recover and were more flattened, they maintained their adhesion to the surface of the culture dish (Fig. 3C).

### BEI-9 downregulates cyclin D1

Progression through the cell cycle is regulated by cyclins and their CDKs. Regulation of cyclin-CDK activity is controlled by synthesis and degradation of the cyclins. Cyclin D1, a regulator involved in the G1-S transition of cells, is an oncogene (27). Since it regulates cell cycle progression, its expression could be affected by treatment with BEI-9, partly accounting for lack of cell cycle progression in treated cells. To this end, the levels of cyclin D1 protein were assessed in the control and treated cells by immunoblotting. Also assessed were the levels of cyclin A, which regulates both G1-S and G2-M transitions. BEI-9 treatment decreased the expression of cyclin D1, as well as that of cyclin a (Fig. 3D), indicating that the ‘freeze’ effect of BEI-9 is mediated by inhibition of cell cycle progression caused by inhibition of the expression of cyclins that drive the transitions.

Similar to the effect for SW480, BEI-9, at 5 and 10 *µ*M, decreased the viability of HCT116 colon cancer cells, as determined by MTS assays (Fig. 3E), and stopped their proliferation (Fig. 3F).

### BEI-9 inhibits NF-κB signaling pathway

Since the multifunctional transcription factor NF-κB is a regulator of cyclin D1 (28), its effects on NF-κB signaling in SW480 cells exposed to BEI-9 were determined. To this end, NF-κB reporter SW480 cells (SW-NFL) stably transduced with a construct containing NF-κB-response elements linked to the luciferase gene as a reporter were used. As demonstrated previously, these cells activate NF-κB in response to TNFα and to some cancer chemotherapeutic drugs (23). As a response to NF-κB activation, these cells express increased amounts of luciferase, which can be measured by luminescence assays.

**Figure 4:**
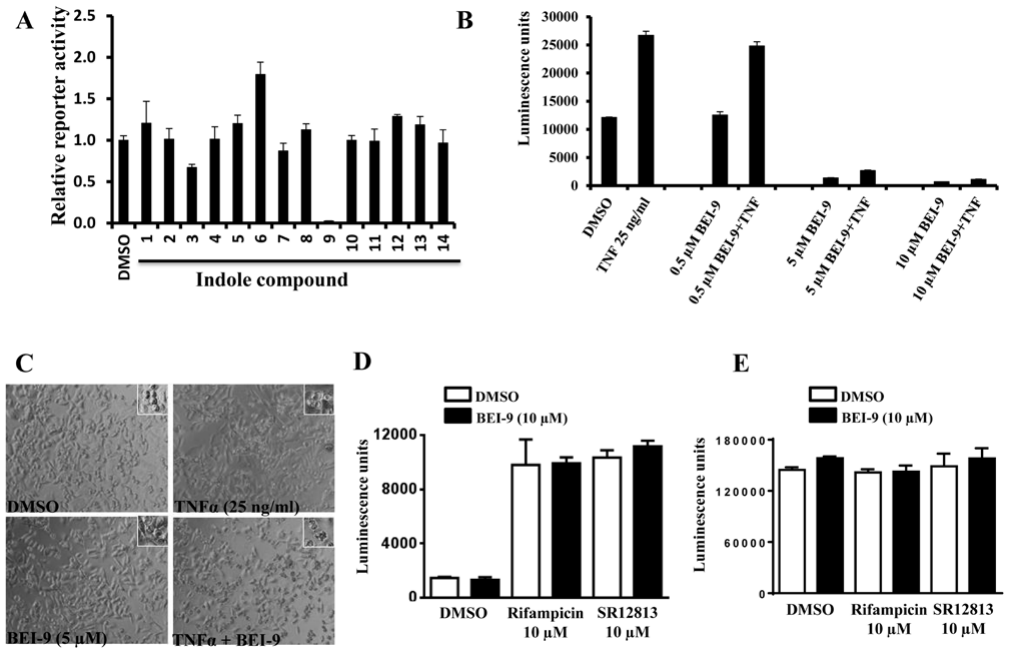
BEI-9 suppresses basal and induced NF-κB activation. (A) SW480 cells engineered to report intracellular activation of NF-κB signaling pathway were exposed to dimethyl sulfoxide (DMSO) or with each of the 14 indole compounds. Basal reporter activity, measured relative to DMSO-treated cells is shown. (B) Reporter activity in SW480 NF-κB reporter cells was induced by treating the cells with 25 ng/ml of TNFα. The effects of DMSO and BEI-9 (0.5, 5, or 10 *µ*M) on TNFα-induced NF-κB reporter activation were measured and plotted as luminescence units. (C) Morphology of SW480 cells exposed to DMSO, TNFα (25 ng/ml), BEI-9 (5 *µ*M), or a combination of TNFα and BEI-9. (d) hepg2 cells were transiently transfected with human pregnane X receptor (hPXR) and luciferase-linked PXR target reporter CyP3a4-luc. At 24 h after transfection, the cells were exposed to DMSO or human PXR agonists (rifampicin or SR12813) for 20 h. BEI-9 (10 *µ*M) or the vehicle control (DMSO) was added to the cells 5 min prior to measuring the luciferase activity using the Neolite Luciferase reporter gene assay. BEI-9 did not directly inhibit the activity of the luciferase enzyme in reporter assays. (E) Cells were similarly treated as in C. DMSO or BEI-9 was added to the cells 5 min prior to measuring cell viability using the CellTiter-Glo Luminescent Cell Viability assay, which measures ATP availability in the cells.

BEI-9 and the other 13 compounds were assessed for their effects on the basal levels of luciferase activity. Equal numbers of SW480-NFL cells were seeded in 96-well plates and exposed to DMSO or to the test compounds. Luciferase activity was measured at 24 h after the treatment. Among the 14 compounds, only BEI-9 reduced the reporter activity (Fig. 4A). Then the possibility that cytokine-induced NF-κB activation is blocked by BEI-9 was determined. Since we previously established that these cells are responsive to TNFα, an inducer of the NF-κB pathway, and to various chemotherapeutic drugs (23), TNFα was used to activate NF-κB in these reporter cells. Cells were exposed to TNFα (25 ng/ml) and to 0.5, 5 or 10 *µ*M BEI-9. At 5 or 10 *µ*M, BEI-9 abolished the activation of NF-κB by TNFα (Fig. 4B). The difference between the effects of 0.5 and 5 *µ*M BEI-9 was <10-fold, showing that, for SW480 cells, effective concentrations of BEI-9 are in a low micromolar range. Although, as single agents, neither of the compounds caused cell death, the combination of TNFα (25 ng/ml) and BEI-9 (5 *µ*M) resulted in the appearance of cells with membrane blebs, which are a characteristic of apoptosis (Fig. 4C). Thus, in addition to inhibiting NF-κB activation, BEI-9 added to TNFα-treated cells may divert TNF receptor-initiated signaling toward apoptosis.

Luciferase reporter assays are dependent on the activity of the luciferase enzyme to catalyze the conversion of luciferin to oxyluciferin in the presence of ATP and oxygen, generating light in the process. Therefore, compounds that directly interfere with the enzyme activity should be distinguished from those that inhibit the signaling activity inside the cells. To test this, the PXR-luciferase reporter system expressed in hepg2 cells was used, and BEI-9 (10 *µ*M) was added to the cells 5 min before measuring luciferase. ATP-dependent cell viability was measured with CellTiter-Glo kits, to determine if BEI-9 competes with cellular ATP, which is required for luciferase activity. The results from both assays (Fig. 4D and E) suggest that, at bioactive concentrations, BEI-9 does not inhibit luciferase directly or indirectly by competing with ATP.

To rule out the possibility that BEI-9 directly inhibits luciferase enzyme activity, the possibility that BEI-9 affects the luciferase activity in HepG2 cell-based luciferase reporter gene assays was assessed. hPXR is a ligand-dependent nuclear receptor that regulates the expression of drug-metabolizing enzymes, including cytochrome p450 (CYP3A4) (29). HPXR transactivation assays were accomplished with HepG2 cells transiently transfected with hPXR and CYP3A4-luc, in which the expression of luciferase was controlled by the hPXR-regulated CYP3A4 promoter. The human PXR agonists, rifampicin and SR12813, induced PXR transactivation of CYP3A4 promoter activity (Fig. 4D). BEI-9 (10 *µ*M) did not affect the luminescence either under basal (DMSO) or stimulated (rifampicin or SR12813) conditions (Fig. 4D), showing that it does not directly inhibit luciferase activity.

**Figure 5:**
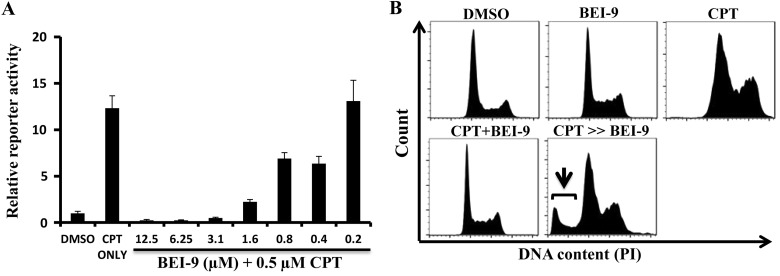
Effect of BEI-9 on induced reporter activity. (A) NF-κB activity in SW480 reporter cells was induced by treating the cells with camptothecin (CPT) (0.5 *µ*M) for 24 h in the presence or absence of the indicated concentrations of BEI-9. Relative reporter activity, normalized to dimethyl sulfoxide (DMSO)-treated cells is shown. (B) Cell cycle profiles of parental SW480 cells exposed to DMSO, BEI-9, CPT, BEI-9 and CPT combined (CPT+BEI-9) for 24 h, or 24 h of CPT followed by 24 h of only BEI-9 (CPT>>BEI-9) are shown. The short arrow points to the sub-G1 population of cells resulting from the sequential treatment. The x-axes of histograms in B indicate propidium iodide (PI) staining intensity, representing the DNA content of cells, whereas the y-axes indicate cell counts.

We previously demonstrated that camptothecin (CPT), a drug used to treat various types of cancers, activates NF-κB in SW480 cells at peak concentrations of 0.5–1 *µ*M (23). To examine the possibility that BEI-9 suppresses the drug-induced NF-κB response in these cells, SW480 reporter cells were exposed to CPT (0.5 *µ*M) and to varying concentrations (0.2–12.5 *µ*M) of BEI-9. BEI-9 inhibited the NF-κB response >50% at concentrations >0.8 *µ*M (Fig. 5A). Although CPT and BEI-9, separately or together, did not induce cell death in these cells, as determined by flow cytometry, sequential treatment of CPT for 24 h followed by BEI-9 for 24 h resulted in the appearance of a distinct sub-G1 population, an indication of cell death (Fig. 5B).

BEI-9 was well tolerated by nude mice at intraperitoneal doses up to 10 mg/kg, but 100 mg/kg was toxic, causing death within 24 h (Table II). A dose of 50 mg/kg, although not as lethal, resulted in loss of body weight and noticeable discomfort to the mice, requiring termination. Doses of 1 and 5 mg/kg did not result in body weight loss, nor did they induce any noticeable change in physical state or behavior. The most obvious gross pathology after repeated intraperitoneal injections was irritation of the peritoneum in the 10 mg/kg dose group, resulting in localized peritoneal adhesions and moderate weight loss. Histopathological analyses of the tissues collected from kidneys, liver, heart, spleen, pancreas, lungs, and intestines did not show pathological abnormalities for doses <10 mg/kg.

## Discussion

In the present study, BEI-9 was characterized as an inhibitor of cell proliferation. It was more potent than I3C, which has anticancer activities (1,2). The mechanisms involved in its anti-proliferative and anti-NF-κB signaling activities remain to be determined. Nevertheless, since NF-κB activation is implicated in carcinogenesis and in reducing sensitivity to anticancer drugs, BEI-9 should be investigated in combination with drugs, such as CPT and docetaxel, which activate NF-κB (23,30–32). BEI-9 was found to be an inhibitor of cell proliferation but did not cause cell death, as evaluated by microscopy and cell cycle analysis. The bioactivity of BEI-9 appears to depend on inhibiting the progression of cells through the cell cycle. Part of this effect may be through enhanced degradation or reduced production of cyclins, which are cell cycle regulators. The possibility of BEI-9 acting as a metabolic inhibitor of cell growth, resulting in cells ‘frozen’ at the phase in which they existed at the time of treatment, needs further examination, particularly in view of the involvement of tryptophan, an indole, in cellular metabolism.

The effects of combinations of CPT and BEI-9 appear to be dependent on the sequence of treatments. This is in accordance with the different mechanisms and dynamics of actions of TNFα and CPT; the slow CPT-induced cellular effects could be overcome by the ‘freeze’ effects of BEI-9, and the rapid receptor effects of TNFα could be modulated by BEI-9 as a second step. However, when CPT was given time to act on the cells, subsequent addition of BEI-9 led to cell death. Further examination of the dynamics of such interactions is needed to identify the mechanisms of BEI-9 interaction with other anticancer agents.

Of note, necrostatins, similar compounds with the indole backbone, are regulators of necroptosis, a form of cell death (33). The cellular targets for necrostatins are the RIPK1 and IDO proteins (34–36), which have potential applications in inflammatory diseases and neuroprotection (37,38). Further research is needed to determine whether common signaling proteins are targeted by necrostatins and BEI-9. Given the similar interference with inflammatory signaling by necrostatins and BEI-9, the latter should be tested for neurovascular and cardiovascular protection.

Since BEI-9 did not have noticeable physical or pathological effects in nude mice at doses below 10 mg/kg, subsequent studies with mice can be designed with 10 mg/kg as the upper limit of dosage. Calculated for a 2-ml volume of mouse blood, the highest tolerable dose we administered to mice (10 mg/kg) corresponds to ~10 *µ*M, well above the effective concentrations that had biological effects such as NF-κB inhibition. Furthermore, since nude mice were used in these experiments, the potential effects of BEI-9 on the immune system or cells thereof need to be evaluated.

## Figures and Tables

**Table I:** Fourteen indole compounds tested for bioactivity on cancer cells at a concentration of 50 *µ*M.

Chemical name	Designation
5-Methoxyindole	Indole-1
5-Chloroindole-3 carboxaldehyde	Indole-2
Indole-5-carboxaldehyde	Indole-3
1-Methylindole-2-carboxaldehyde	Indole-4
Indole-4-carboxylic acid	Indole-5
Ethyl-5-hydroxy-2-methylindole-3-carboxylate	Indole-6
5-Bromoindole-3-acetic acid	Indole-7
Methyindole-3-carboxylate	Indole-8
3-(2-Bromoethyl) indole	BEI-9
Indole-3-carboxylic acid	Indole-10
Indole-3-carboxyladehyde	Indole-11
6-Methoxyindole	Indole-12
5-Hydroxy-indole-3-acetic acid	Indole-13
Indole-3-carbinol	Indole-14

**Table II:** Summary of the experiments to determine a safe dose range for BEI-9 in nude mice.

Dosage group	No. of animals	No. of once-a-week injections	Gross pathology	Histopathology (major findings)	Remarks
Control	5	6	None		
Vehicle (DMSO)	5	6	None		
1 mg/kg	10	6	None		
5 mg/kg	10	6	None		
10 mg/kg	5	6	Peritoneal localized adhesions		Adhesions noted at necropsy
50 mg/kg	2	2	Weight loss, discomfort	Hepatocyte vacuolation, swelling and some degenerative changes	Terminated
100 mg/kg	1	1	Acute toxicity within 24 h	Not performed	Lethal
